# Effects of Banana Flour on Some Physicochemical, Textural, Bioactive, and Sensory Properties of Gluten‐Free Cookie

**DOI:** 10.1002/fsn3.4756

**Published:** 2025-01-17

**Authors:** Kevser Karaman, Hasan Pinar, Beyza Ciftci, Mahmut Kaplan

**Affiliations:** ^1^ Department of Agricultural Biotechnology, Faculty of Agriculture Erciyes University Kayseri Türkiye; ^2^ Department of Horticulture, Faculty of Agriculture Erciyes University Kayseri Türkiye; ^3^ Department of Field Crops, Faculty of Agriculture Erciyes University Kayseri Türkiye

**Keywords:** Banana flour, bioactivity, gluten‐free cookie, texture

## Abstract

The present study aimed to investigate the effects of banana flours obtained from different banana cultivars (Grand Nain (GN), Azman (AZ), and Erdemli (ER)) on some basic physicochemical, bioactive, textural, and sensory properties of the gluten‐free cookie samples by the simplex lattice mixture design approach. Incorporating banana powder into cookie samples resulted in significant changes in the parameters studied. The banana flours’ total dietary fiber and total starch levels ranged between 1.37% and 3.43% and 34.82% and 41.38%, respectively. Erdemli type bananas (ER) were distinguished by their high dietary fiber and resistant starch content as well as their superior bioactive properties. Total phenolic content and antiradical activity of the cookie samples enriched with banana flour were in the range of 376–600 mg GAE/kg, while the antiradical activity values ranged between 8.64% and 59.42%. The hardness of the cookie samples also ranged between 1664.5 and 2605.9 g. According to the results of the optimization, 98.5% ER and 1.5% GN flour mixture could yield the highest response scores. As a conclusion, gluten‐free cookies can be enriched by using banana flour, especially ER‐type ones, to increase the bioactive properties and overall acceptability.

## Graphical abstract

In this study, three different banana types were used in the formulation of gluten‐free cookies. Using the simplex lattice mixture design, fifteen cookie samples were manufactured, and the effect of banana variety on the final product quality was analyzed. Erdemli‐type bananas, which have prominent bioactive properties among the banana cultivars, produced the most sensory favorite cookies.

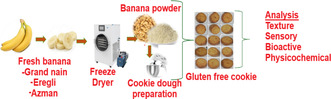

## 1 Introduction

Banana (*Musa paradisiaca*) is a commonly consumed climacteric fruit that is mainly produced in tropical and subtropical developing countries. Silva, Barbosa Junior, and Barbosa (2015) reported that bananas are the fourth most demanded food after rice, wheat, and corn. Bananas are rich in appreciable amounts of vitamins B and C, and they are also a good source of potassium and calcium (Kumar et al. 2012; Ranjha et al. 2022). Banana, especially green banana, the form of the unripe banana, is also very rich in resistant starch type 2 (Cano et al. 1997; de la Torre‐Gutiérrez, Chel‐Guerrero, and Betancur‐Ancona 2008). Banana is a special fruit because it is generally consumed as ripe due to high sugar content and also unripe, which provides high resistant starch intake (Falade and Oyeyinka 2015; Islam et al. 2024). In general, it is preferred to be consumed as ripe by the people, but it is a very functional ingredient as unripe, which can be used as a fortification agent. For the ripe banana, high amounts of fruit are lost during commercialization because of deficient postharvest handling. To prevent the loss of the ripe banana, they can be dried and used in some food formulations as an alternative approach (Ambrose and Naik 2016). Gluten‐free (GF) products are produced generally for patients suffering from celiac disease. It is defined as an autoimmune response showing symptoms including inflammation of the small intestine. Celiac patients cannot consume some foods containing gluten present in wheat, barley, and rye. The only treatment method for this disease is to avoid foods including gluten in their structure (Schuppan, Dennis, and Kelly 2005). GF cereal‐based products generally include starch in high quantity and low dietary fiber levels and no gluten (Hernández‐Aguirre et al. 2019). For that reason, the fortification possibilities of GF products using different natural ingredients have a crucial impact on the GF products industry and also celiac patients. There are many studies about the enrichment of GF products by using different fortification agents (Guadalupe‐Moyano et al. 2022; Olawoye and Gbadamosi 2020; Gamlath 2008; Radoi et al. 2015; Juarez‐Garcia et al. 2006; Mir, Bosco, and Shah 2019; Olaimat et al. 2023).

Around the world, cookies are widely consumed and are healthy source of energy for people of all ages. They are inexpensive, have a long shelf life, and come in a variety of tastes and shapes. Additionally, GF formulations with superior quality and sensory qualities can be used to manufacture them (Martínez et al. 2021). Gluten network formation is not required, and starch gelatinization is the main determinant of cookie texture (Hamdani, Wani, and Bhat 2020). They make up the largest category of bakery snacks due to their affordability, flavor, crispness, and storability. They are also seen to be a good way to give customers nutrients (Mohammadi et al. 2022). Ostermann‐Porcel et al. (2017) investigated the effect of okra powder on GF cookies, and okra's addition raised the amount of fiber and protein. Additionally, hardness values of cookies increased while their whiteness index decreased. In another study, various combinations of date seed flour, modified starch, and chestnut flour were used to create a GF rice flour‐based cookie, and the preferred samples contained 20% date seed flour, 30% chestnut flour, and 0.9% modified starch (Mohammadi et al. 2022).

In the current work, three different banana cultivars (Grand Nain, Azman, and Erdemli) were dried, powdered, and used to enrich the GF cookie formulation. To see the effect of cultivar types, fifteen cookie recipes were created according to the simplex lattice mixture design approach, and an optimization study was conducted to reveal the best cultivar type for the desired and high‐bioactive GF cookie production.

## 2 Materials and Methods

### 2.1 Materials

Three different banana cultivars (Erdemli (ER), Grand Nain (GN), and Azman (AZ)) were procured from Mersin (Türkiye). They were harvested in mature form, and they were freeze‐dried by a lyophilizer (TM25, Techmech, İstanbul, Türkiye) immediately and powdered using a grinder. All banana powder samples were stored at −18°C until use. The cookie ingredients (gluten‐free flour mix, margarine, cream, sugar, baking powder, and egg) were provided by Sofra Degirmencilik Food Co. (Kayseri, Türkiye).

### 2.2 Methods

#### 2.2.1 Characterization of Banana Cultivars

Proximate composition (dry matter, ash, protein, and fat content) of the cookie samples was characterized according to the methodology of AOAC (2005). Megazyme Resistant Starch Assay (K‐RSTAR, Megazyme International Ireland Ltd. Co. Wicklow, Ireland) was used to characterize the resistant, non‐resistant, and total starch content of the banana powder sample (Kaplan, Yüksel, and Karaman 2021; Kardeş et al. 2021). Major sugar profiles (fructose, glucose, and sucrose) were characterized by using the HPLC‐RID system. (Emaga et al. 2007).

For the determination of the mineral composition of the banana powder samples, about 0.5 g of the sample was supplemented with 10 mL of a nitric + perchloric acid mixture and subjected to wet digestion until a final volume of 1 mL. After that, the solutions were diluted with distilled water, and ICP‐OES spectrometer (Perkin‐Elmer, Optima 4300 DV, ICP/OES, Shelton, CT, USA) was used to determine Al, B, Ca, Cr, Cu, Fe, K, Mg, Mn, Na, Ni, P, Pb, S, and Zn (Mertens 2005).

#### 2.2.2 Manufacturing of Gluten‐Free Cookies

Gluten‐free (GF) cookies enriched with different banana powders produced using different banana cultivars were manufactured according to the mixture design approach tabulated in Table 1. Before starting the cookie production, firstly, the binary and ternary banana flour mixtures were prepared according to the proportions given in Table 1. After that, a standard cookie dough was prepared with basic ingredients such as 180 g of gluten‐free flour (Sofra Değirmencilik Food Co, Kayseri, Türkiye), 25 g shortening, 77 g margarine, 23 g cream, 57 g powdered sugar, 1.4 g baking powder, and 35 g whole egg. They were weighed as basic cookie ingredients, and a 50 g banana flour mixture (Table 1) was incorporated into this basic cookie recipe. All ingredients were mixed well for 15 min using a mixer, and the cookie dough was rested for 10 min. Then the dough was shaped using a cookie shaper and placed on the cooking tray. Before cooking, they were rested for 2 min under stretch film, and then the cookies were baked at 170°C for 15 min. After baking the samples, the cookies were cooled and stored for further analysis.

**TABLE 1:** Experimental design used to prepare the banana flour mixture.

Runs	Component 1 (%)	Component 2 (%)	Component 3 (%)
	A: ER	B: GN	C: AZ
1	100.0	0.0	0.0
2	0.0	0.0	100.0
3	0.0	0.0	100.0
4	50.0	50.0	0.0
5	50.0	0.0	50.0
6	16.7	16.7	66.7
7	0.0	100.0	0.0
8	66.7	16.7	16.7
9	33.3	33.3	33.3
10	0.0	50.0	50.0
11	50.0	50.0	0.0
12	16.7	66.7	16.7
13	100.0	0.0	0.0
14	50.0	0.0	50.0
15	0.0	100.0	0.0

Abbreviations: AZ, Azman; ER, Erdemli; GN, Grand Nain.

#### 2.2.3 Characterization of Gluten‐Free Cookies

##### 2.2.3.1 Color

The color parameters (L*, a*, and b*) of the GF cookie samples were measured using a colorimeter (CR‐400 Konica Minolta, Tokyo, Japan). Color was expressed in CIELAB as L* (lightness), a* (redness), and b* (yellowness), and the color measurements were performed on the surface of the cooked cookie sample. Using the following equations, chroma [Equation (1)] and browning index [Equations (2) and (3)] values were also calculated (Khoozani, Bekhit, and Birch 2019; Nasrin, Noomhorm, and Anal 2015). All measurements were repeated five times with two repetitions.

(1) $$\text{Chroma}=\sqrt{a^{2}+b^{2}}$$

(2) $$\text{Browning Index}=\frac{100\left(x-0.31\right)}{0.17}$$

where *x* is calculated as follows:

(3) $$x=\frac{a*+1.75L*}{5.645L*+a*-3.01b*}$$

##### 2.2.3.2 Textural Analysis

Textural properties of GF banana flour‐added cookies were determined using a Texture Analyzer (TA. HD. Pluss Stable Micro Systems, Godalming, UK) equipped with a 3‐point rigid apparatus and a 100 kg loading cell. Hardness of the cookie samples was measured as a textural parameter. For this purpose, textural analysis operation parameters were set as follows: pre‐test speed: 1 mm/s, test speed: 3 mm/s, post‐test speed: 10 mm/s, the mode as distance having 25 mm set values and trigger force was 50 g. All measurements were repeated five times with two repetitions.

##### 2.2.3.3 Bioactive Properties

GF cookies enriched with different banana flour obtained from different cultivars were subjected to phenolic extraction before starting to analyze bioactivity. For this purpose, 1 g of banana powder or cookie samples were mixed with 9 mL of acetone and mixed by vortex. Then the samples were placed in the water bath and shaken for one hour for the extraction. At the end of the duration, the tubes were centrifuged at 7500 g for 10 min at +4°C. Then the supernatant was filtered by a 0.45‐μm syringe filter and used as the material for total phenolic content and antiradical activity analysis.

Total phenolic content of the samples was determined using the method suggested by Singleton and Rossi (1965) with some modifications. For this purpose, 200 μL of the extract was mixed with 1800 μL of distilled water. Then 1 mL of diluted (1/10) Folin–Ciocalteu reagent and, after 1 min, 2 mL of sodium carbonate (2% w/v) was added into all tubes. The samples were incubated for 2 h at room temperature and in dark conditions. At the end of the incubation, the absorbance values of the samples were recorded at 765 nm using a UV–vis spectrophotometer (Shimadzu UV–vis 1800, Japan). Total phenolic content of the samples was calculated as mg GAE/kg sample using a calibration curve.

DPPH radical scavenging activity of the samples was determined as described by He et al. (2016). A 100 μL of the extract sample was mixed with 3900 μL of DPPH radical solution in methanol (2 mM) and mixed well using a vortex. After the incubation of the samples at room conditions in a dark place for 30 min, the absorbance values were recorded at 517 nm by a spectrophotometer (Shimadzu UV–vis 1800, Japan). DPPH radical scavenging capacity was calculated as % inhibition using the following Equation (4):

(4) $$\%\mathrm{Inh}=100-\left[\left({\mathrm{Abs}}_{\text{control}}-{\mathrm{Abs}}_{\text{sample}}\right)/{\mathrm{Abs}}_{\text{control}}\right]\times 100$$

##### 2.2.3.4 Sensory Analysis

Sensory analysis was performed by a panel group that consisted of 10 males and 10 females from the students and staff of the Faculty of Erciyes University. At the beginning of the study, the panel members were informed about the samples, and the analysis was started. All cookie samples were served in a random order, and a hedonic‐scaled evaluation form (1–9 scale) was given for the samples. The panelists were asked to clean their mouth by drinking spring water between the samples. The cookie samples were evaluated in terms of some basic quality parameters such as color, taste, odor, texture, mouth sense, sweetness, and overall preference.

### 2.3 Statistical Analysis

For the statistical evaluation of the results, the SAS 8.0 statistical package program was used. In the data obtained from the analysis results, the effect of the factors on the results was determined by variance analysis (*p* < 0.05), and multiple comparisons and group averages were compared by LSD. Design‐Expert Software Version 7.0 (Stat‐Ease Inc., Minneapolis, USA) was used to calculate the regression coefficients of linear, quadratic, and interaction terms for each output parameter and apply the simplex lattice mixture design. Also, the same software was used to perform variance analysis and the optimization process.

## 3 Results and Discussion

### 3.1 Some Physicochemical Properties of the Banana Cultivars

**FIGURE 1:**
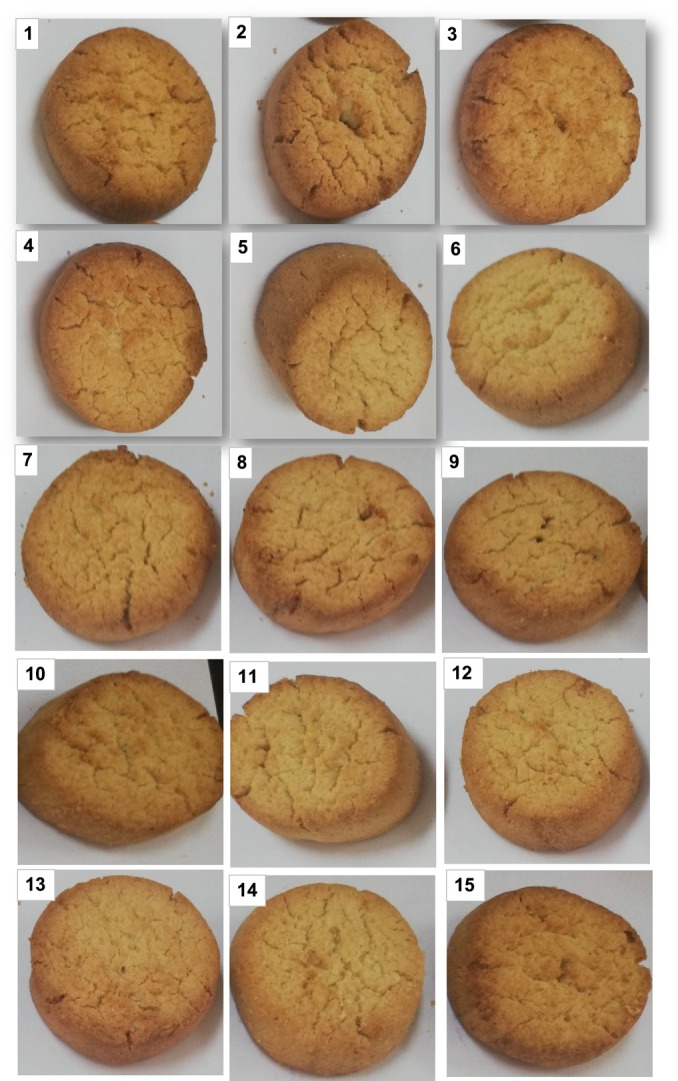
Gluten‐free cookie samples enriched with different banana cultivars.

In the current study, three different banana cultivars were freeze‐dried, powdered, and used as a fortification agent in the production of GF cookies. The manufactured GF cookie samples were characterized in terms of color, bioactive properties, and textural and sensory characteristics. Figure 1 illustrates the images of the manufactured cookie samples. To determine the effect of banana cultivar, basic parameters were modeled using simplex lattice mixture design to observe the effect of banana type on the studied response. Table 2 shows the change in basic physicochemical properties of the banana powders used for the fortification of GF cookies. As is seen, dry matter levels ranged between 16.61% and 30.20%, and the cultivar of ER banana showed the highest dry matter content while the other two‐banana cultivars showed similar results. For the lipid and protein content, AZ had the highest lipid content (4.01%) and GN had the highest protein level (5.10%). Dietary fiber levels of the samples were in the range of 1.37%–3.43%, and ER cultivar also showed the highest fiber level. Starch profile of the banana samples was also characterized and resistant starch which behaves like a dietary fiber was in the range of 2.24%–8.41% and ER cultivar had the higher resistant starch content compared to GN and AZ (*p* < 0.05). Resistant starch of the used ripened banana was determined to be lower compared to the literature results of unripe green bananas. Resistant starch is known as the sum of starch and products of starch degradation not absorbed in the small intestines of healthy individuals, and for that reason, it has a reduced calorie level and shows similar physiological effects to dietary fibers (Juarez‐Garcia et al. 2006). Rodríguez‐Ambriz et al. (2008) reported that the green unripe banana is rich in resistant starch and non‐processed food. It was reported that the green banana flour had 70%–80% starch of the dry weight (Campuzano, Rosell, and Cornejo 2018), and Bi et al. (2017) informed that the resistant starch in the green banana flour is type 2, showing the resistance to the hydrolysis. da Mota et al. (2000) performed comparative research on different cultivars of banana, and they reported that the water levels of the banana flour ranged between 3.91% and 6.17%, and protein and fat levels of the samples were reported to be in the range of 2.5%–3.3% and 0.33%–0.82%, respectively. All banana flour showed quite high starch content, and the highest starch level was measured as 76.5% while the lowest starch level was 61.3%. Dietary fibers were reported as soluble and insoluble, and they ranged between 2.10%–3.05% and 4.10%–12.56%, respectively. de Angelis‐Pereira et al. (2016) also reported that the crude fiber of the banana pulp flour was 3.8%. Khoza, Kayitesi, and Dlamini (2021) reported that the lipid and protein levels of the green banana flour ranged between 0.42%–0.85% and 3.60%–4.33%, respectively. As is seen, the proximate composition of the banana flours showed significant differences depending on the banana cultivar type. In the same study, the starch content of the banana flour samples also ranged between 61.3% and 76.5%. In another study, Bi et al. (2017) reported that the lipid, protein, and starch content of the flour samples was in the range of 0.32%–0.57%, 2.90%–4.59%, and 78.19%–81.82%, respectively. Khoozani, Bekhit, and Birch (2019) also studied the effects of different drying conditions on the starch content, thermal properties, and some of the physicochemical parameters of whole green banana flour, and they reported that the protein, lipid, and carbohydrate contents of the banana flour samples ranged between 3.97%–4.17%, 0.92%–0.93% and 84.61%–85.26%, respectively. Yani, Arief, and Mulyanti (2013) characterized the local banana, and they reported that the protein ranged between 2.55% and 5.58%, while the lipid and crude fiber levels were in the range of 0.43–0.58 and 0.52%–1.90%, respectively. It was concluded that the proximate composition of the banana showed significant differences depending on the cultivar. Similar differences were detected for the major sugar profile of the samples. As is seen in Table 2, fructose, glucose, and sucrose contents of the three banana flour samples were tabulated. Sucrose was the dominant sugar ranging between 8.97% and 15.31%, and the sucrose‐rich banana flour was AZ and GN. Fructose and glucose content of the samples were in the range of 4.72%–5.23% and 5.61%–5.88%, respectively, for the samples. Also, the proximate composition of the banana is quite dependent on the ripening stages of the fruits. Gamlath (2008) reported that the effect of ripening stages on some physicochemical parameters of the banana and the protein, lipid, and dietary fiber levels were in the range of 3.1%–3.9%, 2.3%–3.1%, and 2.4%–2.7%, respectively. The reported values by Gamlath (2008) were found to be quite similar to the results of the current work. Total phenolic content of the banana powder samples was also determined, and it was concluded that the banana variety affected the phenolic concentration significantly (*p* < 0.05). The highest total phenolic content was determined for the ER cultivar (7262 mg GAE/kg), while the lowest one was for the banana cultivar of AZ. Al Amri and Hossain (2018) investigated the bioactivity of two banana genotypes and reported that the total phenolic content was in the range of 1317.2–3862.2 mg GAE/kg for Salalah ripe bananas and 1551.4–3072.2 mgGAE/kg for Pilipino‐type bananas.

**TABLE 2:** Some physicochemical properties of different types of banana powders.

Parameters (%)	GN	AZ	ER	LSD
Fat	3.28^b^	4.01^a^	2.54^c^	0.71**
Protein	5.10^a^	4.26^b^	3.01^c^	0.6**
Dietary fiber	1.37^b^	1.47^b^	3.43^a^	0.23**
Resistant starch	2.34^b^	2.24^b^	8.41^a^	0.36 **
Non‐resistant starch	34.53^a^	32.58^b^	32.97^b^	1.05**
Total starch	36.87^b^	34.82^c^	41.38^a^	1.23**
Fructose	5.23^a^	4.72^b^	4.75^b^	0.31**
Glucose	5.88^a^	5.39^a^	5.61^b^	0.33^a^
Sucrose	14.22^b^	15.31^a^	8.97^c^	0.93**
Total phenolic content (mg GAE/kg)	1740.6^b^	1585.9^c^	7262.5^a^	0.78**

*Note:* Small superscript letters in each row show statistical significance (*p* < 0.05).

Abbreviations: AZ, Azman; ER, Erdemli; GN, Grand Nain.

Table 3 shows the main mineral composition of three different banana cultivars. As is seen from the table, the major and dominant mineral for all banana flour was potassium (K), in which the level ranged between 6868 and 12,222 mg/kg. GN was found to be the richest in K compared to other samples. Second, the most abundant element present in the banana flour was Mg for all samples. Mg was in the range of 1755–2119 mg/kg, and also GN showed the highest Mg level. The minor elements for all banana flour samples were Cr, Mn, and Ni, having levels of 1.92–2.59 mg/kg, 3.55–5.1 mg/kg, and 3.05–4.43 mg/kg, respectively. All banana flours also had a high concentration of phosphorus (P) and calcium (Ca). Gamlath (2008) reported that potassium was the main mineral of banana pulp, and the level of potassium ranged between 323 and 347 mg/100 g depending on the ripening stage. Pareek (2016) reviewed the nutritional and biochemical properties of banana cultivars, and he reported that the banana is very rich in potassium, and an average‐sized banana contained K in the range of 450–467 mg. Wall (2006) reported that the K content of the banana collected from Hawaii was 3306 mg/kg for fresh bananas, and the Mg content of the banana was 351 mg/kg, while Fe, Cu, and Mn were the minor elements for the banana. K level was reported to be 5.09 mg/g for the banana (fw). In another study, Khoza, Kayitesi, and Dlamini (2021) investigated some characteristic properties of green banana flour, and they reported the mineral composition of the different cultivars of green banana flour. As similar to the results of the current work, they reported that the major element for five different banana cultivars was potassium (K), and its concentration ranged between 2900.9–10330.3 mg/kg. The second major element was reported as Mg for the banana samples, and its level was in the range of 324–1001 mg/kg. Zn, Cu, Fe, and Mn were the trace elements for the banana flour with the levels of 0.18–0.93, 0.25–0.50, 1.33–2.88, and 0.48–3.20 mg/100 g, respectively.

**TABLE 3:** Mineral composition of different types of banana powders (mg/kg).

Elements	GN	ER	AZ
Al	14.24^a^	11.675^b^	10.4^c^
B	19.735^a^	14.73^c^	18.28^b^
Ca	780.45^a^	735.39^a^	618.16^b^
Cd	0.17^a^	0.19^a^	0.15^b^
Co	0.095^a^	0.09^c^	0.085^b^
Cr	2.375^b^	1.925^c^	2.59^a^
Cu	203.785^a^	156.36^b^	147.48^b^
Fe	124.9^a^	84.9^b^	71.42^b^
K	12222.93^a^	6868.63^c^	10789.99^b^
Mg	2119.99^a^	1832.07^b^	1755.65^c^
Mn	3.55^b^	5.115^a^	3.745^b^
Na	502.19^a^	406.785^b^	364.14^c^
Ni	4.43^a^	3.345^b^	3.05^b^
P	1324.83^a^	1027.31^c^	1211.57^b^
Pb	11.66^a^	9.57^b^	8.9^b^
S	699.86^a^	434.21^c^	558.14^b^
Zn	55.22^a^	42.47^b^	40.83^b^

*Note:* Small superscript letters in each row show statistical significance (*p* < 0.05).

Abbreviations: AZ, Azman; ER, Erdemli; GN, Grand Nain.

### 3.2 Color and Textural Properties of the Gluten‐Free Cookie Samples

Regarding the color characteristics of the GF cookies enriched with three different banana flours, L*, a*, and b* values of the cookie samples were measured, and using these values, chroma and browning index values of the samples were calculated. According to the simplex lattice mixture design, fifteen cookie samples containing different types of banana flours were manufactured, and their color properties are tabulated in Table 4. As is seen, brightness (L*) values of the samples ranged between 54.07 and 64.63, and the highest L* value was measured for the cookie sample produced according to run 11, while the lowest was for the sample produced based on run 1. The redness (a*) and yellowness (b*) values of the cookie samples were in the range of 9.37–13.99 and 31.44–37.60, respectively. Chroma, which is the function of redness and yellowness values of the samples, ranged between 32.81 and 40.12. The highest C value was determined for the cookie sample added with only the AZ cultivar, while the lowest value was observed for the cookie sample added with only the GN cultivar. Cornejo and Rosell (2015) reported that the chroma (C) close to zero was interpreted to indicate subdued colors, whereas high chroma values were interpreted to specify a more vibrant color. As is seen, the cookie samples showed a more vibrant color. Browning index values of the samples ranged between 75.87 and 125.91, and the sample showing the highest and lowest chroma value also showed the highest and lowest browning index, respectively, and a very high and significant correlation (*r* = 0.98, *p* < 0.05) was observed between chroma values and browning index.

**TABLE 4:** Bioactive characteristic, hardness, and color properties of the cookie samples enriched with banana flour.

Runs	L*	a*	b*	C	BI	Hardness (g)	TPC (mg GAE/kg)	ARA (%)
1	54.07 ± 3.35	12.85 ± 0.79	36.25 ± 1.26	38.46 ± 1.03	120.29 ± 5.54	2001.4 ± 125.5	546.61 ± 17.6	52.25 ± 2.15
2	57.40 ± 2.10	13.51 ± 0.73	32.56 ± 0.96	35.25 ± 0.86	97.59 ± 2.57	2381.2 ± 145.2	508.81 ± 61.7	21.15 ± 2.54
3	54.63 ± 2.53	13.99 ± 1.13	37.60 ± 1.36	40.12 ± 1.20	125.91 ± 6.35	2104.3 ± 158.5	500.00 ± 19.0	23.65 ± 3.52
4	56.48 ± 0.78	12.78 ± 0.51	33.90 ± 0.39	36.23 ± 0.42	103.74 ± 8.55	2016.4 ± 196.3	498.05 ± 47.4	52.87 ± 2.56
5	62.63 ± 3.56	10.02 ± 1.93	31.54 ± 1.61	33.09 ± 1.77	79.65 ± 4.57	1936.1 ± 225.9	391.97 ± 47.2	45.49 ± 3.52
6	62.18 ± 1.29	10.06 ± 0.60	33.38 ± 0.39	34.86 ± 0.50	86.05 ± 3.62	2226.7 ± 236.4	411.45 ± 23.7	48.16 ± 1.25
7	62.11 ± 3.14	11.56 ± 1.17	35.15 ± 0.90	37.00 ± 1.01	93.64 ± 2.69	2161.9 ± 154.1	420.92 ± 23.2	9.39 ± 0.99
8	59.75 ± 3.93	11.26 ± 0.66	31.72 ± 0.57	33.66 ± 0.65	86.93 ± 3.45	2081.9 ± 158.2	472.38 ± 17.8	58.67 ± 2.22
9	61.61 ± 1.67	11.12 ± 0.81	33.22 ± 0.93	35.03 ± 0.82	87.93 ± 3.20	2605.9 ± 194.5	441.83 ± 104.3	51.19 ± 2.96
10	61.46 ± 1.55	11.14 ± 0.64	35.11 ± 0.86	36.83 ± 0.75	94.35 ± 2.95	1750.0 ± 147.7	385.00 ± 96.9	59.42 ± 3.56
11	64.63 ± 2.97	10.39 ± 0.84	32.45 ± 1.21	34.07 ± 1.03	79.43 ± 2.10	1664.5 ± 102.3	600.00 ± 167.0	33.46 ± 2.10
12	60.32 ± 1.87	10.96 ± 0.10	34.96 ± 0.33	36.64 ± 0.23	96.08 ± 2.52	2102.5 ± 178.5	376.00 ± 165.5	22.26 ± 1.95
13	60.53 ± 1.71	12.34 ± 1.13	32.76 ± 0.41	35.01 ± 0.77	90.08 ± 2.65	1855.8 ± 165.4	540.00 ± 57.2	58.23 ± 2.96
14	64.32 ± 2.80	9.37 ± 1.10	31.44 ± 0.82	32.81 ± 0.96	75.87 ± 1.95	2152.6 ± 199.5	396.00 ± 87.2	39.64 ± 2.00
15	58.53 ± 0.31	13.27 ± 0.12	36.67 ± 0.37	39.00 ± 0.25	109.46 ± 3.54	2206.3 ± 251.1	416.00 ± 96.9	8.64 ± 0.56

Abbreviations: ARA, antiradical activity (%); BI, browning index; C, chroma; TPC, total phenolic content (mg GAE/kg).

**FIGURE 2:**
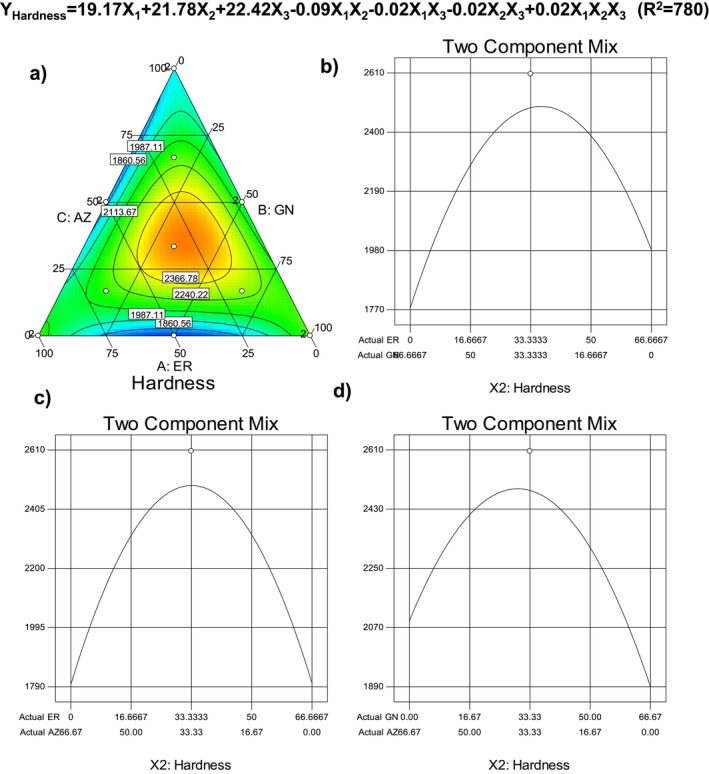
Change in hardness of the gluten‐free cookie depending on the banana flour type.

Hardness values of the sample ranged between 1664.5 and 2605.9 g for the cookie samples, and the lowest hardness value was recorded for the cookie sample produced with the mixture of ER and GN (50:50 w/w) banana flours, while the highest hardness was measured for the sample added with the triple mixture of the banana flours at equal ratios (33:33:33% w/w). The hardness of the cereal‐based products is an important quality parameter for consumer preference because the textural characteristics of the foods have an important role in the eating quality (Mancebo, Picón, and Gómez 2015). For that reason, the effects of the flour type and ratio on the hardness values of the cookie samples were characterized by using the modeling with simplex lattice mixture design. The change in the hardness values of the cookie samples is illustrated in Figure 2 as a binary and ternary plot. As is seen, hardness values of the cookie samples decreased toward the vertex of the plot and showed a considerable increment toward the middle of the plot. So, using the mixture of all banana cultivar flours as enrichment agents in GF cookies caused a significant increase in the hardness of the final products. The linear effect of the mixture variable on the hardness values of the samples was found to be insignificant while the mixture effect of the flours was determined to be very significant (*p* < 0.01; Table 5). The interaction effect of banana flours was monitored as very similar to each other (Figure 2a–c). The selected model was also significant, showing that the selected cubic model could be used to explain the flour mixture effect on the hardness of the cookie samples (*p* < 0.001; Table 5). The constructed regression model is also given in Figure 2, and it can be stated that the model could be used to estimate the hardness values of the cookies depending on the flour samples because it has an acceptable determination coefficient (*R* ^2^ = 0.780). Mancebo, Picón, and Gómez (2015) studied the effects of different flour types on some quality characteristics of GF cookies, and they reported that the hardness of the cookies was significantly affected by the flour type, and they attributed the reason for the increased hardness of the cookies to the high protein content of the different flours used. Farris and Piergiovanni (2009) reported that the reason for the increased hardness in cookies was that fiber and protein complexation occurred during cooking at high temperatures and time combinations. Also, the sugar content of the samples was effective on the hardness values of the samples because the conversion of sugar into solution caused a harder glass‐like state when the cookies are cooled. Singh et al. (2002) also monitored that the hardness of the cookies increased with the increase of sugar level.

**TABLE 5:** ANOVA table for the effects of mixture variable on some quality parameters.

Source	df	Hardness (Qubic model)		TPC (Quadratic model)			ARA (Quadratic model)		Overall acceptability (Quadratic model)	
		Mean square	*F*	df	Mean square	*F*	Mean square	*F*	Mean square	*F*
Model	6	101388.9	4.71^a^	5	11613.4	12.19^a^	778.00	11.44^a^	0.324	7.92^a^
Linear mixture	2	57736.3	2.68	2	8332.2	8.75^a^	1066.97	15.69^a^	0.414	10.15^a^
AB	1	68144.3	3.17	1	6139.4	6.45^b^	117.95	1.73	0.033	0.81
AC	1	3086.8	0.14	1	25253.7	26.51^a^	18.44	0.27	0.023	0.56
BC	1	187570.0	8.72^b^	1	7803.9	8.19^b^	1601.92	23.56^a^	0.726	17.76^a^
ABC	1	441533.5	20.53^a^	—						
Residual	8	21511.1	0.45	9	952.6	0.78	67.99	2.43	0.041	0.84
Lack of Fit	3	12268.0	4.71	4	823.9	12.19	101.07	11.44	0.037	7.92
Pure Error	5	27056.92		5	1055.5	8.75	41.53	15.69	0.044	10.15
Cor Total	14	101388.9		14		6.45		1.73		

^a^ *p* < 0.01.

^b^ *p* < 0.05.

### 3.3 Bioactivity of the Gluten‐Free Cookie Samples

**FIGURE 3:**
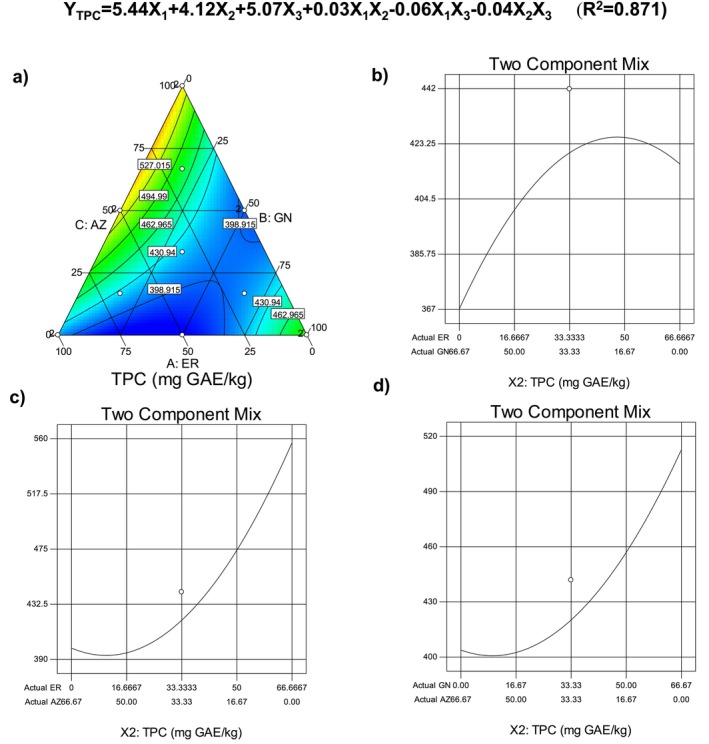
Change in total phenolic content of the gluten‐free cookie depending on the type of banana flour.

**FIGURE 4:**
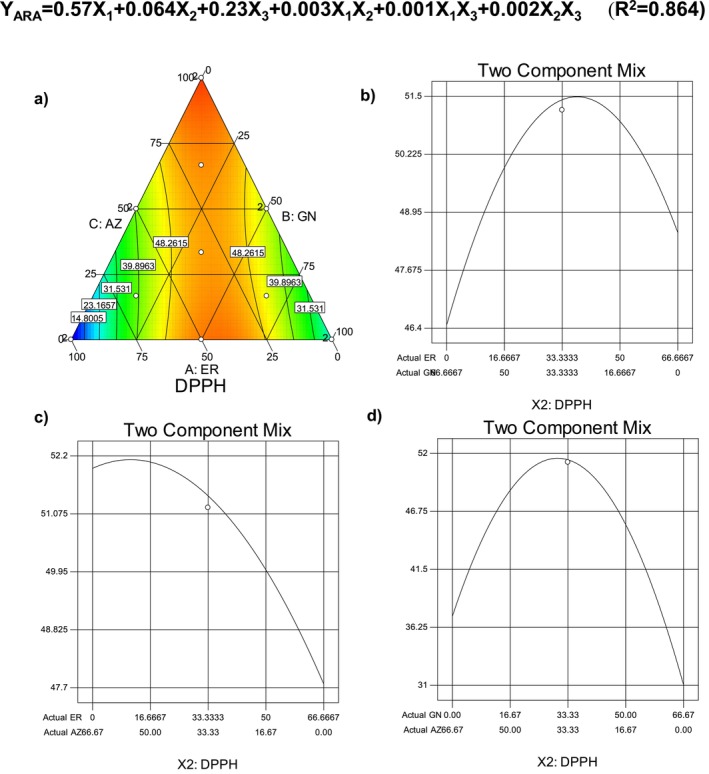
Change in antiradical activity of the gluten‐free cookie depending on the banana flour type.

For the bioactive characterization of the cookie samples, total phenolic content (TPC) and antiradical activity (ARA) of the samples were determined. As is seen in Table 4, TPC levels of the samples were in the range of 376–600 mg GAE/kg, while the antiradical activity values were in the range of 8.64%–59.42%. The highest TPC was determined for the cookie sample added with the mixture of ER and GN cultivar flours, and the lowest TPC was for the sample produced with the mixture of all cultivar flours prepared by ER, GN, and AZ at the levels of 16.7%, 66.7%, and 16.7%, respectively. For the ARA values, the high radical scavenging capacity was observed for the sample added with GN and AZ cultivar flours at the level of 50:50 (%, w/w), and the lowest antiradical activity was observed for two cookie samples produced with the addition of only GN cultivar flour (100%). Pareek (2016) reported that the banana fruit is rich in phytochemicals, and especially unripe bananas contain a high quantity of tannins, which are responsible for the unpleasant astringent taste, and their level significantly decreases with the ripening of the fruit. It was also reported that the phenolics in the banana structure are responsible for the oxidative browning reaction when the fruit is cut. As is known, phenolic substances present in many foods, especially in fruit and vegetables, have a significant role in the prevention and protection from several degenerative diseases in humans (Vieira et al. 2011). Due to the importance of the bioactivity of the foods, the change in TPC and ARA showing the bioactive performance of the cookie samples depending on the banana cultivar type, TPC and ARA were modeled using a simple lattice mixture design approach. Figures 3, 4 illustrate the change in TPC and ARA values of the cookie samples. As is seen, the TPC of cookie samples increased toward the vertex of ER and GN, but the highest phenolics were determined for the sample having a high level of banana flour obtained from the ER cultivar. Increase in the AZ cultivar caused lower total phenolic content for the cookie sample. The linear mixture effect of the flour was found to be very significant (*p* < 0.01; Table 5), and also a significant effect was observed for the interaction between the cultivar flours (*p* < 0.05; Table 5). As is seen from the binary interaction graph, the decrease of AZ flour in the binary mixture resulted in a sharp and significant increase in the total phenolic content of the cookie samples (Figure 3b,c). The selected quadratic model was also significant (*p* < 0.01), and the constructed regression model was acceptable for the total phenolic content due to a high determination of coefficient (*R* ^2^ = 0.871). Radoi et al. (2015) produced gluten‐free pasta enriched with banana flour, and they reported that the addition of banana flour provided a significant increase in total phenolic content of the final samples.

The change in antiradical activity of the cookie samples is given in Figure 4. As is seen, ARA activity of the samples decreased toward the vertex of GN and AZ cultivar flours while there was a clear increase toward the edge of ER and also the vertex of the AZ and GN. The linear mixture effect of the flours was determined to be significant (*p* < 0.05; Table 5), and the interaction between GN and AZ cultivars was also effective on the antiradical activity of the cookie samples (*p* < 0.05; Table 5). As is seen from Figure 4d, using GN and AZ together showed quite high antiradical performance. For the interaction between ER and AZ, a decrease in AZ and an increase in ER caused a sharp decrease in the ARA activity, as did the interaction between GN and AZ. The selected quadratic model was also significant, and the constructed regression model showed a high estimation capacity for the antiradical activity of the samples due to a high coefficient of determination (*R* ^2^ = 0.864). Wang, Zhang, and Mujumdar (2012) reported that the addition of 50% green banana flour improved the nutritional value, especially fiber, minerals, polyphenols, and the antioxidant capacity of the snacks. Radünz et al. (2021) reported that the radical scavenging activity of muffins enriched with green banana flour was 54.9%, while the only green banana flour showed a 65.58% inhibition percentage.

### 3.4 Sensory Characteristics of the Gluten‐Free Cookie Samples

**FIGURE 5:**
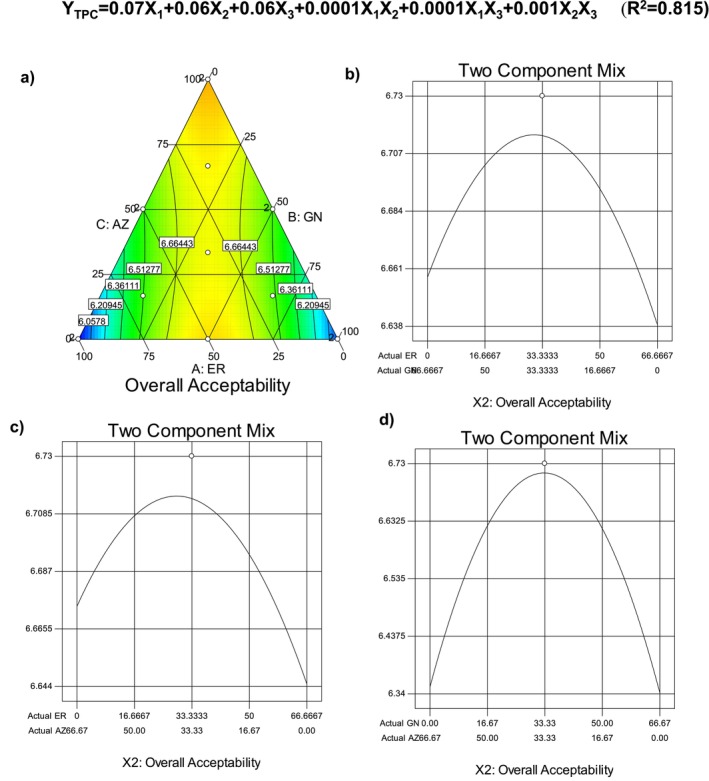
Change in overall acceptability of the gluten‐free cookie depending on the banana flour type.

Within the context of sensory analysis, the color, taste, odor, texture, mouthsense, sweetness, and overall preference scores of the cookie samples enriched with different cultivars of banana flours are tabulated in Table 6. Color values ranged between 5.82 and 6.82, taste values ranged between 5.45 and 7.00, odor values ranged between 5.55 and 6.91, texture scores ranged between 5.82 and 7.36, mouthsense scores ranged between 5.91 and 7.00, sweetness scored ranged between 5.91 and 7.00, and the overall preference score showing the general evaluation for all sensory parameters was in the range of 5.90–7.00. To make a general evaluation for the sensory scores of the cookie samples, the change in overall acceptability of the samples was modeled using simplex lattice mixture design. The change in overall preference scores of the cookie samples is illustrated as a binary and ternary plot in Figure 5. As is seen, in general, the overall preference scores increased toward the vertex of ER and the edges of GN and AZ. Linear mixture effect on the overall preference scores was determined to be very significant (*p* < 0.01; Table 5). For the binary mixtures, at the equal mixtures, the cookies gained high overall preference scores, but the sole usage of GN and AZ in the formulation decreased the preference scores because the lowest acceptability was recorded for the cookie sample produced with the use of only GN or AZ cultivar flour. But their interactive usage increased the scores significantly (*p* < 0.05; Table 5). The selected quadratic model was significant, and the constructed regression model showed quite a high determination of coefficient (*R* ^2^ = 0.815). Radünz et al. (2021) produced gluten‐free muffins added with green banana flour, and they reported that the overall scores of the muffins were quite high because the acceptability index of the muffins prepared with green banana flour was 84.5%. Detchewa et al. (2021) produced rice cookies added with different levels of unripe banana flour and reported that the usage of banana flour higher than 80% caused a significant decrease in the color, taste, and overall preference scores because the texture of the cookies became hard and the products smelled like raw banana, and high banana flour levels caused darkening (lower L* value), less reddish (lower a* value), and less yellowish (lower b* value) color. Udachan et al. (2022) produced gluten‐free brown rice pasta formulated with green matured banana flour and defatted soy flour, and they reported that the brown rice pasta had a positive effect on sensory analysis up to 30% replacement.

**TABLE 6:** Sensory scores of the cookie samples enriched with banana flour.

	Color	Taste	Odor	Texture	Mouthsense	Sweetness	Overall preference
1	6.55 ± 1.21	6.45 ± 1.21	6.00 ± 1.61	7.18 ± 1.40	6.45 ± 1.21	7.00 ± 1.00	6.91 ± 0.94
2	5.82 ± 1.78	5.82 ± 1.89	6.18 ± 1.83	6.82 ± 0.98	6.09 ± 2.07	5.91 ± 2.02	6.00 ± 2.41
3	6.27 ± 1.42	5.64 ± 1.69	5.55 ± 1.75	6.09 ± 0.94	5.91 ± 1.87	5.82 ± 1.66	5.91 ± 1.51
4	6.55 ± 1.21	6.64 ± 1.50	5.73 ± 1.79	6.55 ± 1.37	6.64 ± 1.86	6.82 ± 1.25	6.73 ± 1.42
5	6.45 ± 1.37	6.55 ± 1.57	6.27 ± 2.00	6.91 ± 1.51	6.55 ± 1.75	6.82 ± 1.72	6.64 ± 1.63
6	6.82 ± 1.40	6.18 ± 1.08	5.91 ± 1.22	6.45 ± 1.37	6.64 ± 1.29	6.82 ± 0.98	6.55 ± 0.93
7	6.55 ± 1.37	6.64 ± 0.92	6.18 ± 1.47	6.27 ± 1.10	6.82 ± 0.87	7.00 ± 1.18	5.90 ± 0.67
8	6.55 ± 1.04	7.00 ± 1.48	6.91 ± 1.04	7.36 ± 1.63	6.73 ± 1.49	6.91 ± 1.45	7.00 ± 1.55
9	6.73 ± 1.74	6.64 ± 1.12	5.73 ± 1.27	6.27 ± 1.10	6.64 ± 1.12	6.82 ± 0.75	6.73 ± 1.10
10	6.55 ± 1.92	6.82 ± 1.66	6.36 ± 1.21	6.18 ± 1.40	7.00 ± 1.67	6.82 ± 1.47	6.82 ± 1.47
11	6.64 ± 1.36	6.36 ± 1.63	6.18 ± 1.60	6.73 ± 1.56	6.45 ± 1.51	6.55 ± 1.51	6.27 ± 1.56
12	6.64 ± 1.12	6.18 ± 1.94	6.09 ± 1.70	6.73 ± 1.27	6.00 ± 2.10	6.73 ± 2.05	6.27 ± 2.05
13	6.55 ± 1.13	6.64 ± 1.57	6.64 ± 1.36	6.73 ± 1.19	6.27 ± 1.56	6.82 ± 1.33	6.64 ± 1.43
14	6.36 ± 1.75	5.45 ± 1.97	6.45 ± 2.21	6.55 ± 1.57	6.00 ± 2.14	6.55 ± 1.97	6.27 ± 1.95
15	6.27 ± 1.49	6.09 ± 1.45	5.82 ± 1.83	5.82 ± 1.94	5.91 ± 2.07	5.91 ± 1.87	6.00 ± 1.90

### 3.5 Multiple Response Optimization of the Mixture Variables

To reveal the optimum banana flour type and concentrations, multiple response optimization was conducted. For this aim, a desirability function approach was used, and in the optimization procedure, total phenolic content, antiradical activity, and overall acceptability scores were maximized. According to the optimization results, the best flour mixture should be composed of 98.5% ER and 1.5% GN to obtain the maximum total phenolic content, antiradical activity, and overall acceptability scores. The desirability function value was calculated as 0.839 for this optimization. In addition to that, using sole GN banana flour in the production of GF cookies would be caused by very low scores for the studied response.

## 4 Conclusion

Different banana cultivars, namely Grand Nain (GN), Azman (AZ), and Erdemli (ER), showed significant differences when compared to each other, and the Erdemli cultivar had superior biofunctionality because of the quite high resistant starch and also dietary fiber level. The preferred GF cookies by panelists also showed differences in terms of banana flour in the samples. In general, the cookie samples having ER cultivar showed high total phenolic content and antiradical activity. Using banana flour increases the hardness values of the samples slightly. Multiple response optimization also revealed that the best mixture levels showing the highest total phenolic content, antiradical activity, and overall acceptability scores for the cookie could be obtained by using the Erdemli cultivar for almost all of the cookie recipe. For further research, the Erdemli cultivar could be characterized in detail, and it can be evaluated in different GF recipes.

## Author Contributions

**Kevser Karaman:** formal analysis (supporting), writing – review and editing (lead). **Hasan Pinar:** data curation (equal), resources (equal), writing – review and editing (equal). **Beyza Ciftci:** formal analysis (lead). **Mahmut Kaplan:** conceptualization (equal), data curation (equal), writing – review and editing (equal).

## Conflicts of Interest

The authors declare no conflicts of interest.

## Data Availability

The data that support the findings of this study are available in the manuscript.

## References

[fsn34756-bib-0001] Al Amri, F. S. , and M. A. Hossain . 2018. “Comparison of Total Phenols, Flavonoids and Antioxidant Potential of Local and Imported Ripe Bananas.” Egyptian Journal of Basic and Applied Sciences 5, no. 4: 245–251.

[fsn34756-bib-0002] Ambrose, D. C. , and R. A. V. I. N. D. R. A. Naik . 2016. “Development of a Process for Utilization of Banana Waste.” International Journal of Research in Applied, Natural and Social Sciences 4, no. 6: 83–88.

[fsn34756-bib-0003] AOAC Official Method 934.01 . 2005. Official Methods of Analysis of AOAC International. 18th ed. Gaithersburg, MD: AOAC International.

[fsn34756-bib-0004] Bi, Y. , Y. Zhang , H. Jiang , et al. 2017. “Molecular Structure and Digestibility of Banana Flour and Starch.” Food Hydrocolloids 72: 219–227.

[fsn34756-bib-0005] Campuzano, A. , C. M. Rosell , and F. Cornejo . 2018. “Physicochemical and Nutritional Characteristics of Banana Flour During Ripening.” Food Chemistry 256: 11–17. 29606425 10.1016/j.foodchem.2018.02.113

[fsn34756-bib-0006] Cano, M. P. , B. de Ancos , M. C. Matallana , M. Cámara , G. Reglero , and J. Tabera . 1997. “Differences Among Spanish and Latin‐American Banana Cultivars: Morphological, Chemical and Sensory Characteristics.” Food Chemistry 59, no. 3: 411–419.

[fsn34756-bib-0007] Cornejo, F. , and C. M. Rosell . 2015. “Physicochemical Properties of Long Rice Grain Varieties in Relation to Gluten Free Bread Quality.” LWT—Food Science and Technology 62, no. 2: 1203–1210.

[fsn34756-bib-0008] da Mota, R. V. , F. M. Lajolo , B. R. Cordenunsi , and C. Ciacco . 2000. “Composition and Functional Properties of Banana Flour From Different Varieties.” Starch‐Stärke 52, no. 2–3: 63–68.

[fsn34756-bib-0009] de Angelis‐Pereira, M. C. , M. D. F. P. Barcelos , R. C. Pereira , J. D. A. R. Pereira , and R. V. de Sousa . 2016. “Chemical Composition of Unripe Banana Peels and Pulps Flours and Its Effects on Blood Glucose of Rats.” Nutrition & Food Science 46, no. 4: 504–516.

[fsn34756-bib-0010] de la Torre‐Gutiérrez, L. , L. A. Chel‐Guerrero , and D. Betancur‐Ancona . 2008. “Functional Properties of Square Banana ( *Musa balbisiana* ) Starch.” Food Chemistry 106, no. 3: 1138–1144.

[fsn34756-bib-0011] Detchewa, P. , P. Prasajak , W. Sriwichai , and A. Moongngarm . 2021. “The Effects of Unripe Banana Flour on Resistant Starch Content and Quality Characteristics of Gluten‐Free Rice Cookies.” Journal of Sustainable Science and Management 16, no. 2: 67–78.

[fsn34756-bib-0012] Emaga, T. H. , R. H. Andrianaivo , B. Wathelet , J. T. Tchango , and M. Paquot . 2007. “Effects of the Stage of Maturation and Varieties on the Chemical Composition of Banana and Plantain Peels.” Food Chemistry 103, no. 2: 590–600.

[fsn34756-bib-0013] Falade, K. O. , and S. A. Oyeyinka . 2015. “Color, Chemical and Functional Properties of Plantain Cultivars and Cooking Banana Flour as Affected by Drying Method and Maturity.” Journal of Food Processing and Preservation 39, no. 6: 816–828.

[fsn34756-bib-0014] Farris, S. , and L. Piergiovanni . 2009. “Optimization of Manufacture of Almond Paste Cookies Using Response Surface Methodology.” Journal of Food Process Engineering 32, no. 1: 64–87.

[fsn34756-bib-0015] Gamlath, S. 2008. “Impact of Ripening Stages of Banana Flour on the Quality of Extruded Products.” International Journal of Food Science & Technology 43, no. 9: 1541–1548.

[fsn34756-bib-0016] Guadalupe‐Moyano, V. , A. Palacios , C. M. Rosell , and F. Cornejo . 2022. “Impact of Drying Methods on Banana Flour in the Gluten‐Free Bread Quality.” LWT‐ Food Science and Technology 168: 113904.

[fsn34756-bib-0017] Hamdani, A. M. , I. A. Wani , and N. A. Bhat . 2020. “Gluten Free Cookies From Rice‐Chickpea Composite Flour Using Exudate Gums From Acacia, Apricot and Karaya.” Food Bioscience 35: 100541.

[fsn34756-bib-0018] He, B. , L. L. Zhang , X. Y. Yue , et al. 2016. “Optimization of Ultrasound Assisted Extraction of Phenolic Compounds and Anthocyanins From Blueberry ( *Vaccinium ashei* ) Wine Pomace.” Food Chemistry 204: 70–76. 26988477 10.1016/j.foodchem.2016.02.094

[fsn34756-bib-0019] Hernández‐Aguirre, M. A. , J. J. Islas‐Hernández , M. E. Sánchez‐Pardo , S. L. Rodríguez‐Ambriz , and P. Osorio‐Díaz . 2019. “Response Surface Methodology for Optimization of Gluten‐Free Bread Made With Unripe Banana Flour.” Journal of Food Measurement and Characterization 13, no. 3: 1652–1660.

[fsn34756-bib-0020] Islam, S. , M. A. S. Miah , M. F. Islam , K. J. Tisa , and M. M. H. Mondol . 2024. “Enzymatic Extraction of Green Banana Resistant Starch for Future Food Preparation: Structural, Physicochemical and Functional Characterization.” Future Foods 9: 100308.

[fsn34756-bib-0021] Juarez‐Garcia, E. , E. Agama‐Acevedo , S. G. Sáyago‐Ayerdi , S. L. Rodriguez‐Ambriz , and L. A. Bello‐Perez . 2006. “Composition, Digestibility and Application in Breadmaking of Banana Flour.” Plant Foods for Human Nutrition 61, no. 3: 131–137. 17048100 10.1007/s11130-006-0020-x

[fsn34756-bib-0022] Kaplan, M. , F. Yüksel , and K. Karaman . 2021. “In Vitro Glycemic Index, Antioxidant Capacity and Some Physicochemical Characteristics of Deep‐Fried Sorghum Based Gluten Free Chips.” Journal of Food Science and Technology 58, no. 10: 3725–3733. 34471296 10.1007/s13197-020-04830-7 PMC8357898

[fsn34756-bib-0023] Kardeş, Y. M. , M. Kaplan , H. Kale , et al. 2021. “Biochemical Composition of Selected Lines From Sorghum (Sorghum Bicolor L.) Landraces.” Planta 254, no. 2: 1–13. 34228194 10.1007/s00425-021-03670-9

[fsn34756-bib-0024] Khoozani, A. A. , A. E. D. A. Bekhit , and J. Birch . 2019. “Effects of Different Drying Conditions on the Starch Content, Thermal Properties and Some of the Physicochemical Parameters of Whole Green Banana Flour.” International Journal of Biological Macromolecules 130: 938–946. 30844459 10.1016/j.ijbiomac.2019.03.010

[fsn34756-bib-0025] Khoza, M. , E. Kayitesi , and B. C. Dlamini . 2021. “Physicochemical Characteristics, Microstructure and Health Promoting Properties of Green Banana Flour.” Food 10, no. 12: 2894. 10.3390/foods10122894 PMC8700615 34945445

[fsn34756-bib-0026] Kumar, K. S. , D. Bhowmik , S. Duraivel , and M. Umadevi . 2012. “Traditional and Medicinal Uses of Banana.” Journal of Pharmacognosy and Phytochemistry 1, no. 3: 51–63.

[fsn34756-bib-0027] Mancebo, C. M. , J. Picón , and M. Gómez . 2015. “Effect of Flour Properties on the Quality Characteristics of Gluten Free Sugar‐Snap Cookies.” LWT—Food Science and Technology 64, no. 1: 264–269.

[fsn34756-bib-0028] Martínez, E. , R. García‐Martínez , M. Álvarez‐Ortí , A. Rabadán , A. Pardo‐Giménez , and J. E. Pardo . 2021. “Elaboration of Gluten‐Free Cookies With Defatted Seed Flours: Effects on Technological, Nutritional, and Consumer Aspects.” Food 10, no. 6: 1213. 10.3390/foods10061213 PMC8228105 34072109

[fsn34756-bib-0029] Mertens, D. 2005. “AOAC Official Method 975.03. Metal in Plants and Pet Foods.” In Official Methods of Analysis, edited by W. Horwitz and G. W. Latimer , 18th ed. Gaithersburg, MD, USA: AOAC International.

[fsn34756-bib-0030] Mir, S. A. , S. J. D. Bosco , and M. A. Shah . 2019. “Technological and Nutritional Properties of Gluten‐Free Snacks Based on Brown Rice and Chestnut Flour.” Journal of the Saudi Society of Agricultural Sciences 18, no. 1: 89–94.

[fsn34756-bib-0031] Mohammadi, M. , N. Khorshidian , M. Yousefi , and A. M. Khaneghah . 2022. “Physicochemical, Rheological, and Sensory Properties of Gluten‐Free Cookie Produced by Flour of Chestnut, Date Seed, and Modified Starch.” Journal of Food Quality 2022, no. 1: 5159084.

[fsn34756-bib-0032] Nasrin, T. A. A. , A. Noomhorm , and A. K. Anal . 2015. “Physico‐Chemical Characterization of Culled Plantain Pulp Starch, Peel Starch, and Flour.” International Journal of Food Properties 18, no. 1: 165–177.

[fsn34756-bib-0033] Olaimat, A. N. , W. M. Al‐Rousan , K. M. Marazeeq , et al. 2023. “Physicochemical and Sensory Characteristics of Gluten‐Free Corn‐Based Biscuit Supplemented With Walnut and Peanut for Celiac Patients.” Journal of the Saudi Society of Agricultural Sciences 22, no. 7: 413–419.

[fsn34756-bib-0034] Olawoye, B. , and S. O. Gbadamosi . 2020. “Sensory Profiling and Mapping of Gluten‐Free Cookies Made From Blends Cardaba Banana Flour and Starch.” Journal of Food Processing and Preservation 44, no. 9: e14643.

[fsn34756-bib-0035] Ostermann‐Porcel, M. V. , N. Quiroga‐Panelo , A. N. Rinaldoni , and M. E. Campderrós . 2017. “Incorporation of Okara Into Gluten‐Free Cookies With High Quality and Nutritional Value.” Journal of Food Quality 2017, no. 1: 4071585.

[fsn34756-bib-0036] Pareek, S. 2016. “Nutritional and Biochemical Composition of Banana (Musa spp.) Cultivars.” Nutritional Composition of Fruit Cultivars: 49–81.

[fsn34756-bib-0037] Radoi, P. B. , E. Alexa , I. Radulov , et al. 2015. “Total Phenolic, Cinnamic Acids and Selected Microelements in Gluten Free Pasta Fortified With Banana.” Revista de Chimie 66, no. 8: 1162–1165.

[fsn34756-bib-0038] Radünz, M. , T. M. Camargo , C. F. P. Nunes , et al. 2021. “Gluten‐Free Green Banana Flour Muffins: Chemical, Physical, Antioxidant, Digestibility and Sensory Analysis.” Journal of Food Science and Technology 58, no. 4: 1295–1301. 33746257 10.1007/s13197-020-04638-5 PMC7925776

[fsn34756-bib-0039] Ranjha, M. M. A. N. , S. Irfan , M. Nadeem , and S. Mahmood . 2022. “A Comprehensive Review on Nutritional Value, Medicinal Uses, and Processing of Banana.” Food Reviews International 38, no. 2: 199–225.

[fsn34756-bib-0040] Rodríguez‐Ambriz, S. L. , J. J. Islas‐Hernández , E. Agama‐Acevedo , J. Tovar , and L. A. Bello‐Pérez . 2008. “Characterization of a Fibre‐Rich Powder Prepared by Liquefaction of Unripe Banana Flour.” Food Chemistry 107, no. 4: 1515–1521.

[fsn34756-bib-0041] Schuppan, D. , M. D. Dennis , and C. P. Kelly . 2005. “Celiac Disease: Epidemiology, Pathogenesis, Diagnosis, and Nutritional Management.” Nutrition in Clinical Care 8, no. 2: 54–69. 16013224

[fsn34756-bib-0042] Silva, A. D. A. , J. L. Barbosa Junior , and M. I. M. J. Barbosa . 2015. “Green Banana Flour as a Functional Ingredient in Food Products.” Ciência Rural 45: 2252–2258.

[fsn34756-bib-0043] Singh, N. , S. Gupta , N. Singh Sodhi , and R. P. Singh . 2002. “Effect of Additives on Dough and Cookie Making Properties of Flour.” International Journal of Food Properties 5, no. 3: 547–562.

[fsn34756-bib-0044] Singleton, V. L. , and J. A. Rossi . 1965. “Colorimetry of Total Phenolics With Phosphomolybdic–Phosphotungstic Acid Reagents.” American Journal of Enology and Viticulture 16: 144–158.

[fsn34756-bib-0045] Udachan, I. , A. Gatade , R. Ranveer , S. Lokhande , G. Mote , and A. K. Sahoo . 2022. “Quality Evaluation of Gluten‐Free Brown Rice Pasta Formulated With Green Matured Banana Flour and Defatted Soy Flour.” Journal of Food Processing and Preservation 46, no. 9: e16448.

[fsn34756-bib-0046] Vieira, F. G. K. , G. D. S. C. Borges , C. Copetti , P. F. Di Pietro , E. da Costa Nunes , and R. Fett . 2011. “Phenolic Compounds and Antioxidant Activity of the Apple Flesh and Peel of Eleven Cultivars Grown in Brazil.” Scientia Horticulturae 128, no. 3: 261–266.

[fsn34756-bib-0047] Wall, M. M. 2006. “Ascorbic Acid, Vitamin A, and Mineral Composition of Banana (Musa Sp.) and Papaya ( *Carica papaya* ) Cultivars Grown in Hawaii.” Journal of Food Composition and Analysis 19, no. 5: 434–445.

[fsn34756-bib-0048] Wang, Y. , M. Zhang , and A. S. Mujumdar . 2012. “Influence of Green Banana Flour Substitution for Cassava Starch on the Nutrition, Color, Texture and Sensory Quality in Two Types of Snacks.” LWT—Food Science and Technology 47: 175–182.

[fsn34756-bib-0049] Yani, A. , R. W. Arief , and N. Mulyanti . 2013. “Processing of Banana Flour Using a Local Banana as Raw Materials in Lampung.” International Journal on Advanced Science, Engineering and Information Technology 3, no. 4: 289.

